# Radium-223 in metastatic castration-resistant prostate cancer: whole-body diffusion-weighted magnetic resonance imaging scanning to assess response

**DOI:** 10.1093/jncics/pkad077

**Published:** 2023-10-03

**Authors:** Chris Parker, Nina Tunariu, Holly Tovey, Roberto Alonzi, Matthew D Blackledge, Gary J R Cook, Sue Chua, Yong Du, Shaista Hafeez, Iain Murray, Anwar R Padhani, John Staffurth, Alison Tree, Helen Stidwill, Jessica Finch, Andra Curcean, Peter Chatfield, Sophie Perry, Dow-Mu Koh, Emma Hall

**Affiliations:** The Royal Marsden NHS Foundation Trust, London, UK; The Institute of Cancer Research, London, UK; The Royal Marsden NHS Foundation Trust, London, UK; The Institute of Cancer Research, London, UK; The Institute of Cancer Research, London, UK; Mount Vernon Cancer Centre, Northwood, UK; The Institute of Cancer Research, London, UK; Cancer Imaging Department and King’s College London and Guy’s and St Thomas’ PET Centre, King’s College London, London, UK; The Royal Marsden NHS Foundation Trust, London, UK; The Royal Marsden NHS Foundation Trust, London, UK; The Royal Marsden NHS Foundation Trust, London, UK; The Institute of Cancer Research, London, UK; The Royal Marsden NHS Foundation Trust, London, UK; Paul Strickland Scanner Centre, Mount Vernon Cancer Centre, Northwood, UK; Velindre Cancer Centre, Cardiff, UK; The Royal Marsden NHS Foundation Trust, London, UK; The Institute of Cancer Research, London, UK; The Royal Marsden NHS Foundation Trust, London, UK; Mount Vernon Cancer Centre, Northwood, UK; The Royal Marsden NHS Foundation Trust, London, UK; The Institute of Cancer Research, London, UK; The Institute of Cancer Research, London, UK; The Institute of Cancer Research, London, UK; The Royal Marsden NHS Foundation Trust, London, UK; The Institute of Cancer Research, London, UK; The Institute of Cancer Research, London, UK

## Abstract

**Background:**

Radium-223 is a bone-seeking, ɑ-emitting radionuclide used to treat men with bone metastases from castration-resistant prostate cancer. Sclerotic bone lesions cannot be evaluated using Response Evaluation Criteria in Solid Tumors. Therefore, imaging response biomarkers are needed.

**Methods:**

We conducted a phase 2 randomized trial to assess disease response to radium-223. Men with metastatic castration-resistant prostate cancer and bone metastases were randomly allocated to 55 or 88 kBq/kg radium-223 every 4 weeks for 6 cycles. Whole-body diffusion-weighted magnetic resonance imaging (DWI) was performed at baseline, at cycles 2 and 4, and after treatment. The primary endpoint was defined as a 30% increase in global median apparent diffusion coefficient.

**Results:**

Disease response on DWI was seen in 14 of 36 evaluable patients (39%; 95% confidence interval = 23% to 56%), with marked interpatient and intrapatient heterogeneity of response. There was an association between prostate-specific antigen response and MRI response (odds ratio = 18.5, 95% confidence interval = 1.32 to 258, *P* = .013). Mean administered activity of radium-223 per cycle was not associated with global MRI response (*P* = .216) but was associated with DWI response using a 5-target-lesion evaluation (*P* = .007). In 26 of 36 (72%) patients, new bone metastases, not present at baseline, were seen on DWI scans during radium-223 treatment.

**Conclusions:**

DWI is useful for assessment of disease response in bone. Response to radium-223 is heterogeneous, both between patients and between different metastases in the same patient. New bone metastases appear during radium-223 treatment.

The REASURE trial is registered under ISRCTN17805587.

Radium-223 is a bone-seeking, ɑ-emitting radionuclide that is approved for use in metastatic castration-resistant prostate cancer. The ALSYMPCA trial showed that radium-223 improves overall survival, quality of life, and time to skeletal events (1).

Radium-223 is used for men with bone metastases from castration-resistant prostate cancer. Assessment of disease response in bone metastases is suboptimal; sclerotic bone lesions cannot be evaluated on computed tomography/Response Evaluation Criteria in Solid Tumors, and standard imaging criteria from the Prostate Cancer Working Group define progression based only on development of new bone lesions, confirmed on a subsequent scan (2). Imaging criteria documenting response and progression in bone metastases could inform treatment strategies, such as timely stopping of ineffective treatment, optimization of dose scheduling according to response, and identifying prognostic markers of response.

Whole-body diffusion-weighted magnetic resonance imaging (DWI), combining standard anatomical sequences with a functional sequence, has emerged as a new imaging method able to document response and progression in bone metastases (3). DWI can visualize and quantify differences in the mobility of water in tissues, with water mobility being impeded to a greater degree in cellular tissues such as cancer; the quantitative parameter, the apparent diffusion coefficient, has been shown to correlate with tumor cellularity in bone metastases (4) and other tumors (5). With effective treatment, bone and soft tissue metastases show an increase in the apparent diffusion coefficient.

We conducted a prospective phase 2 study of radium-223 incorporating whole-body DWI in men with metastatic castration-resistant prostate cancer. We have previously reported on treatment compliance and on fracture risk after treatment (6). We now report on the primary objective, which was to assess disease response on whole-body DWI. Secondary objectives included assessing disease response by circulating biomarkers, including serum markers of bone turnover (alkaline phosphatase [ALP]), prostate-specific antigen (PSA), and circulating tumor cells. We hypothesized that there would be a dose-response relationship for radium-223, so we studied 2 dose levels—55 kBg/kg and 88 kBq/kg—to maximize the range of administered activity of radium-223.

## Methods

REASURE was a phase 2, multicenter, noncomparative, randomized study of radium-223 in men with chemotherapy-naive, bone-only, progressive metastatic castration-resistant prostate cancer incorporating whole-body DWI and positron emission tomography (PET) imaging as well as biological samples, with the primary objective of identifying potential imaging response biomarkers. The PET data will be reported separately. The trial was registered [ISRCTN17805587 (7)], approved by the National Health Service Health Research Authority Committee London–Surrey Borders Research Ethics Committee (14/LO/1385), co-sponsored by the Institute of Cancer Research (ICR) and the Royal Marsden NHS Foundation Trust, and conducted in accordance with the principles of good clinical practice. All the participants provided written informed consent before study entry. The Clinical Trials and Statistics Unit at the ICR (ICR-CTSU; London, UK) coordinated the study.

Patients were identified through oncology clinics at 3 participating sites in the United Kingdom. Inclusion criteria were histologically or cytologically confirmed adenocarcinoma of the prostate with castration-resistant disease, serum PSA of 2 ng/mL or more, ECOG performance status of 0 to 2, life expectancy beyond 6 months, and multiple skeletal metastases (≥2 hotspots) on bone scintigraphy within the previous 12 weeks. Exclusion criteria were visceral disease; lymphadenopathy greater than 1.5 cm in diameter; inadequate hematologic, kidney, or liver function; prior chemotherapy for castration-resistant prostate cancer; and any prior radioisotope therapy.

Eligible participants were allocated to 1 of 2 dose levels—55 or 88 kBq/kg—of radium-223 (1:1 allocation ratio). Treatment allocation was through minimization with a random element; balancing factors included patient weight, total ALP level, and current bisphosphonate use. Dose allocation was not blinded. Patients received treatment with radium-223 at the allocated dose by intravenous administration every 4 weeks for up to 6 cycles. Whole-body DWI scans were done at baseline, at cycles 2 and 4, and 1 month after treatment. Blood tests, including full blood count, routine biochemistry, and PSA, were done at 4-week intervals during radium-223 treatment. During the follow-up period, patients were evaluated every 4 months for 1 year, during which time imaging was done according to routine clinical practice.

Whole-body DWI was performed axially from the skull vertex to midthigh using a free-breathing, single-shot, echo-planar DWI technique on a 1.5 T MR system (Siemens Aera and Avanto systems, Siemens Healthineers, Erlangen, Germany). Imaging parameters included short τ inversion recovery fat suppression (inversion time = 180 ms), partition thickness of 5 mm, matrix size of 150 × 144, partial Fourier transform of 6 of 8, time to echo of 64.8 ms, repetition time of 14.6 s, receiver bandwidth of 1961 Hz/pixel, *b* values of 50 and 900 s/mm^2^, 3-scan trace-weighted diffusion encoding, and a field of view of 400 × 390 mm^2^. The number of signal averages was 4 for each *b* value. In addition, axial volume interpolated breath hold gradient echo, 2-point Dixon T1-weighted MR images with in-phase and opposed-phase echo timing matched to the same imaging field of view and slice thickness as the DWI were acquired using the following imaging parameters: matrix size = 256 × 105, slice thickness = 5 mm, repetition time = 386 ms, time to echo = 4.8 ms, flip angle = 70°, and number of averages = 1.

For each whole-body DWI examination, metastatic bone disease segmentation was performed using OsiriX MD 2016 software (Pixmeo, Bernex, Switzerland). One junior radiologist (A.C., who had less than 3 years of experience) outlined all sites of metastatic bone disease based on all available sequences; the volumes were then checked and modified as necessary by an experienced radiologist (>10 years of experience with whole-body DWI). The outlined segmentations were then transferred to the apparent diffusion coefficient map to record the individual lesions and global (total tumor burden) apparent diffusion coefficient value for each patient at each imaging time point. In addition, up to 5 representative target lesions larger than 2 cm (1 each from the cervical spine, thoracic spine, lumbar spine, thorax, and pelvis) were selected by the same experienced radiologist (N.T.) and their individual apparent diffusion coefficient values recorded for each patient at each imaging time point.

### Statistical methods

The coefficient of repeatability of apparent diffusion coefficient measurement for bone disease on DWI has been reported to be approximately 15% (8), so a relative increase of 30% in the apparent diffusion coefficient was classed as a response. It was estimated that approximately 25% of untreated patients may demonstrate this level of response due to biological variability or chance. Using a 1-sample comparison of a proportion against a fixed threshold—α = .05 (1-sided) and 83% power—36 evaluable patients were required to detect a response rate of 45% and exclude a rate of 25%. A 5% dropout rate was incorporated, resulting in a target sample size of 38 patients. The sample size was also planned to be sufficient to detect a 45% difference in the proportion of patients responding between doses (35% response rate in the 55-kBq/kg group vs 80% in the 88-kBq/kg group), with 80% power and α = .05 (1-sided).

The primary outcome was the response rate in the evaluable population according to whole-body DWI, defined as a 30% increase in global median apparent diffusion coefficient (8), from before treatment to any point after the first injection until the end of cycle 6. Secondary outcomes included qualitative assessment of response according to METastasis Reporting and Data System for Prostate Cancer (MET-RADS-P) (3) criteria and quantitative response, defined as a 30% increase in the mean of the mean apparent diffusion coefficient values for 5 representative target lesions. The latter was added as an exploratory analysis aiming to document the response heterogeneity noticed during the qualitative evaluation. Other secondary outcomes were response according to PSA level (defined as a 50% reduction from baseline pretreatment levels, confirmed more than 3 weeks later), ALP (defined as a 30% reduction from baseline, confirmed at least 4 weeks later), and circulating tumor cells (defined as conversion from ≥5 cells/7.5 mL at baseline to <5/7.5 mL, confirmed by a second consecutive reading obtained at least 4 weeks later; only those patients with ≥5/7.5 mL circulating tumor cell rate at baseline were included), time to PSA progression, time to ALP progression, and overall survival. Data on time to fracture have been previously reported (6).

Analyses were conducted in the evaluable population, defined as patients who were evaluable for the parameters of interest. Patients with ineligibility deviations were discussed with the independent data monitoring committee on a case-by-case basis; those with deviations likely to affect assessment of the primary endpoint were excluded. Dose groups were combined for analysis unless otherwise specified. Response rates are presented with 2-sided 95% confidence intervals (CIs). The 2-sided 90% confidence interval for the primary outcome is also presented, equivalent to a 1-sided 95% confidence interval per the sample size calculation. Agreement between responses according to different imaging modalities was assessed using Cohen κ. The association between responses according to imaging modalities and blood markers was assessed using the Fisher exact test and logistic regression modeling, with adjustment for minimization balancing factors. Receiver operating characteristic analysis was carried out to explore the relationship between continuous global apparent diffusion coefficient change and other markers of response. Time to ALP or PSA progression and overall survival were assessed using standard Kaplan-Meier methods. The association between response and dose level was assessed using logistic regression. The association with dose was also assessed on a per-lesion basis using a multilevel model with random effect for patient and fixed effect for dose group. All tests of statistical significance were 2-sided (unless otherwise stated), and a cutoff of 5% was used to determine significance.

Analyses were conducted using Stata, version 16.1, statistical software (StataCorp LP, College Station, TX) and based on a snapshot of data taken on January 14, 2021. Overall survival data were updated in April 2022.

## Results

**Figure 1.**
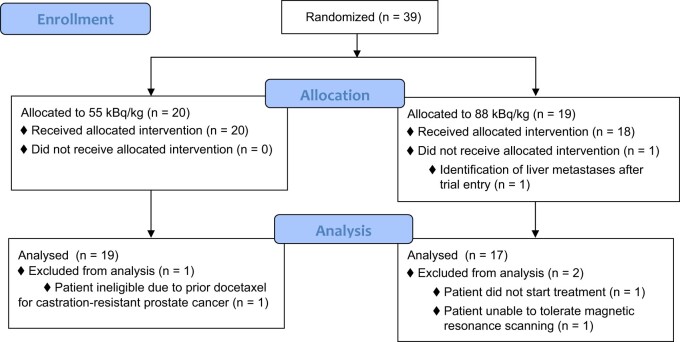
REASURE CONSORT diagram.

Thirty-nine patients were recruited between July 2015 and June 2017. Three patients were excluded from analysis: 1 for detection of liver metastases on baseline imaging, 1 for prior docetaxel for castration-resistant prostate cancer, and 1 who did not tolerate magnetic resonance imaging (MRI) (Figure 1). Baseline patient characteristics for the remaining 36 patients (19 allocated to the 55-kBq/kg group; 17 allocated to the 88-kBq/kg group) are shown in Table 1.

**Table 1.** Demographics and tumor characteristics

	55 kBq/kg (n = 19)	88 kBq/kg (n = 17)	Total (N = 36)
Age at trial entry, median (IQR), y	75.6 (73.0-80.1)	74.5 (7.6-78.1)	75.1 (72.8-79.5)
Ethnicity No. (%)			
British	18 (94.7)	17 (100.0)	35 (97.2)
Other	1^a^ (5.3)	0 (0.0)	1 (2.8)
Weight,^b^ No. (%)			
<80 kg	9 (47.4)	9 (52.9)	18 (50.0)
≥80 kg	10 (52.6)	8 (47.1)	18 (50.0)
ALP,^b^ No. (%)			
<220 U/L	13 (68.4)	16 (94.1)	29 (80.6)
≥220 U/L	6 (31.6)	1 (5.9)	7 (19.4)
PSA level, median (IQR), ng/mL	63.0 (24.0-246.0)	39.0 (15.0-87.1)	43.1 (16.5-167.5)
Extent of disease,^c^ No. (%)			
1	9 (47.4)	8 (47.1)	17 (47.2)
2	5 (26.3)	8 (47.1)	13 (36.1)
3	3 (15.8)	1 (5.9)	4 (11.1)
4	2 (10.5)	0 (0.0)	2 (5.6)
Gleason score (total), No. (%)			
6	1 (5.3)	2 (11.8)	3 (8.3)
7	7 (36.8)	2 (11.8)	9 (25.0)
8	3 (15.8)	4 (23.5)	7 (19.4)
9	8 (42.1)	8 (47.1)	16 (44.4)
Missing	0 (0.0)	1 (5.9)	1 (2.8)
Received prior abiraterone, No. (%)	3 (15.8)	2 (11.8)	5 (13.9)
Received prior enzalutamide, No. (%)	1 (5.3)	2 (11.8)	3 (8.3)
Time since diagnosis, median (IQR), y	3.2 (1.6-5.8)	4.4 (3.0-7.1)	3.9 (2.3-6.7)

a Arab. ALP = alkaline phosphatase; IQR = interquartile range; PSA = prostate-specific antigen.

b Balancing factors for randomization.

c Extent of disease 0 = normal (abnormal because of benign bone disease); extent of disease 1 = fewer than 6 metastatic sites; extent of disease 2 = 6-20 metastatic sites; extent of disease 3 = more than 20 lesions but not a superscan; extent of disease 4 = superscan, defined as diffuse, intense, skeletal uptake of the tracer, with no kidney or background activity.

### Safety

The safety findings were consistent with the known toxicity profile of radium-223. Five serious adverse events were observed, only 1 of which was possibly related to radium-223 (leg pain). No grade 4 or 5 adverse events were reported. Grade 3 adverse events were reported in 17 patients (9 in the 55-kBq/kg group and 8 in the 88-kBq/kg group).

### MRI response

**Figure 2.**
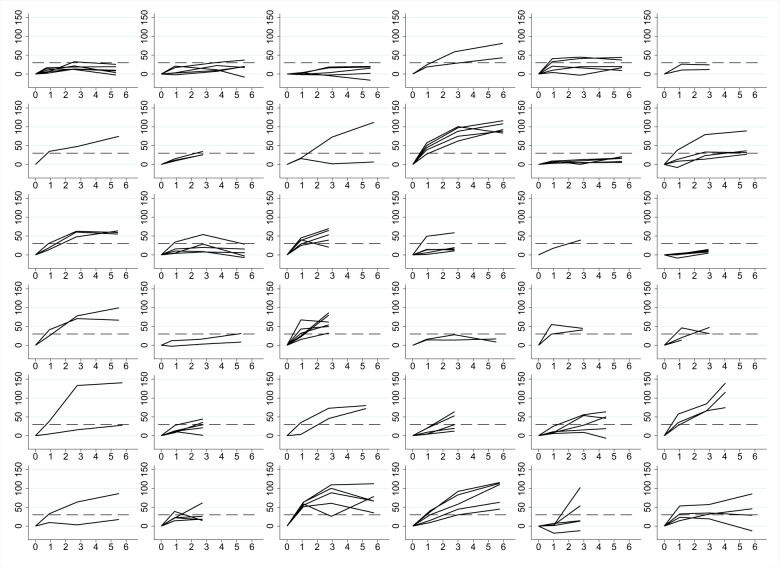
Per-lesion percentage change in mean apparent diffusion coefficient from baseline (0) to cycle 6 of radium-223. Each graph represents data for an individual patient. Each **line** on each graph represents data for an individual lesion.

Disease response on whole-body DWI, defined as a 30% increase in global median apparent diffusion coefficient, was seen in 14 of 36 patients (39%, 95% CI = 23% to 56%, 90% CI = 25% to 54%). For 5 of these patients, response was observed after the first cycle of treatment. Based on a 30% increase in the mean apparent diffusion coefficient of 5 target lesions, response was seen in 23 of 36 patients (64%, 95% CI = 46% to 79%; 90% CI = 49% to 77%). There was marked heterogeneity of response, both between different patients and between different metastases within the same patient (Supplementary Figure 1, available online). This finding is illustrated in Figure 2, which shows the changes in mean apparent diffusion coefficient during treatment for up to 5 bone metastases for each individual patient. Notably, in 26 of 36 patients (72%), new bone metastases, not present on the baseline scan, were seen on MRI during the treatment period.

### Biochemical response

**Figure 3.**
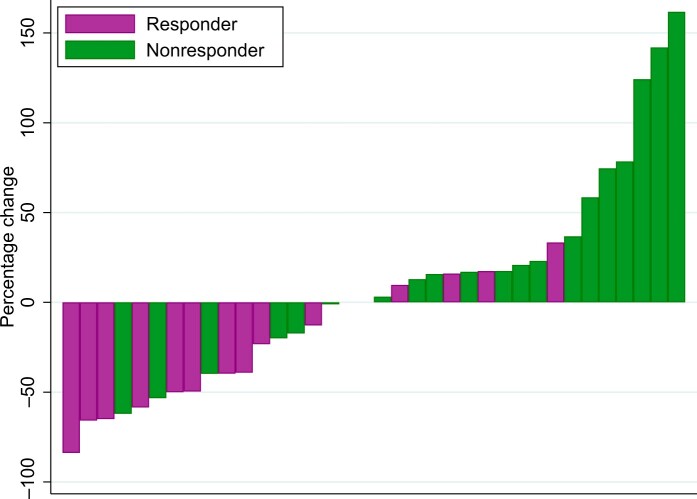
Waterfall plot of best change in prostate-specific antigen, by global apparent diffusion coefficient defined response.

PSA response was seen in 5 of 36 patients (14%). There was an association between PSA response and MRI response (odds ratio = 18.5, 95% CI = 1.32 to 258, *P* = .013). Receiver operating characteristic analysis of global apparent diffusion coefficient as a continuous variable with respect to PSA response gave an area under the curve of 0.90. The best percentage change in PSA from baseline was greater in patients with MRI response than in those who did not respond on MRI (*P* = .004), as shown in Figure 3. Median time to PSA progression was 6.5 months in MRI responders and 4.1 months in MRI nonresponders (*P* = .212). A response in ALP was seen in 24 of 36 patients (67%). No significant association was seen between ALP response and MRI response (*P* = .162).

### Overall survival

Median overall survival in the evaluable population was 27.5 months (95% CI = 14.7 to 37.1). Median overall survival was 30.5 months (95% CI = 11.9 to 51.3) in MRI responders and 17.2 months (95% CI = 7.0 to 37.1) in MRI nonresponders. Table 2 shows the association between overall survival and different measures of MRI imaging response.

**Table 2.** Overall survival, by MRI response

Imaging modality	Hazard ratio	95% Confidence interval	Log-rank *P*
MRI global apparent diffusion coefficient	0.89	0.44 to 1.83	.759
MRI, 5 target lesions	1.29	0.60 to 2.76	.512
MRI qualitative^a^	0.37 (root mean square β = .44)	0.18 to 0.77 (root mean square 0.98 to 5.9)	.006 (root mean square .008)

a Assumption of proportional hazards does not hold for this response definition, so association was also tested using regression of the restricted mean survival times. MRI = magnetic resonance imaging.

### Circulating tumor cell response

Seventeen patients were evaluable for circulating tumor cell response, which was seen in 6 of 17 patients (35%). There was no significant association between MRI response and circulating tumor cell response (odds ratio = 5.1, 95% CI = 0.3 to 93.0, *P* = .256).

### Dose response

**Figure 4.**
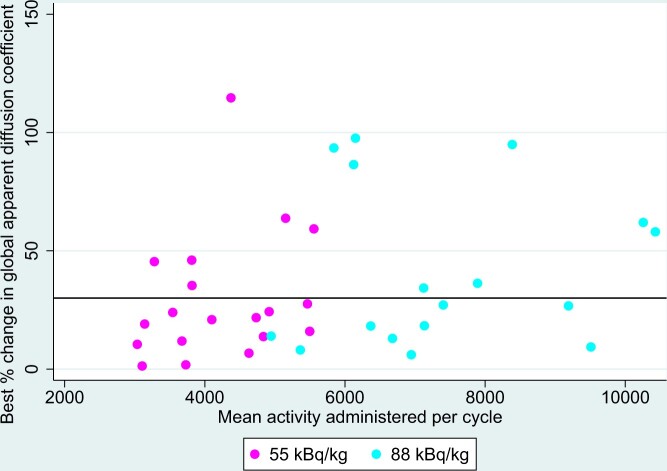
Best percentage change in global apparent diffusion coefficient, by mean activity administered per cycle.

Mean administered activity of radium-223 in kBq per cycle was not associated with global MRI response (*P* = .216) but was associated with MRI response using the 5 target lesion measure (*P* = .007). Figure 4 shows the association between mean administered activity of radium-223 and best change in apparent diffusion coefficient.

**Figure 5.**
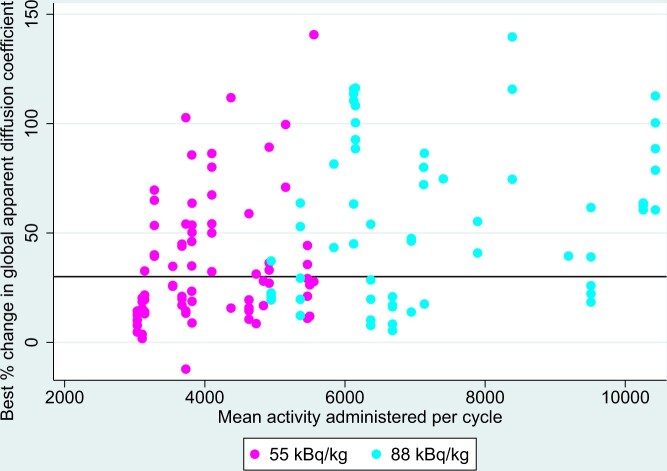
Best percentage change in individual lesion apparent diffusion coefficient, by mean activity administered per cycle.

On a per-lesion analysis, overall, 134 lesions were assessed in 36 patients; MRI response, defined as a 30% increase in mean apparent diffusion coefficient, was seen in 55% (73/134) of patients (Figure 5). MRI response was seen in 34 of 75 (45%; 95% CI = 34% to 57%) of lesions in the 55-kBq/kg group and in 39 of 59 (66%; 95% CI = 53% to 78%) of lesions in the 88-kBq/kg group, giving an odds ratio for response of 6.7 in favor of 88 kBq/kg (95% CI = 1.05 to 42.88).

## Discussion

This study provides evidence that DWI is a useful method for assessment of disease response in bone metastases. It also gives new insights into the mechanism of action of radium-223 in men with metastatic castration-resistant prostate cancer, particularly regarding heterogeneity of response, dose response, and patterns of disease progression.

Baseline whole-body DWI is prognostic of outcomes in a range of cancer types. The apparent diffusion coefficient is thought to provide a measure of tissue cellularity, and it is plausible that more cellular tumors should have worse outcomes. An increase in the apparent diffusion coefficient of bone and soft tissue lesions has been associated with reduction in tumor cellularity following successful therapy. Increasingly, serial whole-body DWI is being used to assess disease response, particularly in relation to bone metastases (eg, MET-RADS-P for prostate cancer, MY-RADS for myeloma) because it is not possible to image response in bone using conventional imaging with computed tomography or Tc99m phosphonate bone scans. Although it is often assumed that changes on whole-body DWI reflect tumor response, there has been a lack of compelling evidence linking imaging changes over time to other measures of disease response and clinical outcomes. In this study, we have shown that whole-body DWI response is associated with PSA response and overall survival. This finding strengthens the rationale for using whole-body DWI to assess response in bone metastases. Whole-body DWI is set to be a valuable tool in the clinic, particularly in patients with bone-only metastases, to document early response, early progression, and heterogenous response without the need for a confirmatory scan, thus enabling a more accurate tailoring of treatment, such as early stopping of ineffective treatment or potentially combinations of systemic therapy with targeted radiation therapy. It will also be useful in clinical research for providing an early outcome measure without the need for long-term follow-up for more established clinical outcomes.

This study is the first to observe the pattern of whole-body DWI response to radium-223 in men with metastatic castration-resistant prostate cancer. We found marked interpatient and intrapatient heterogeneity of response. The ALSYMPCA trial found an overall survival benefit for radium-223, with a median improvement of 4 months, in a relatively unselected population of patients with bony metastatic castration-resistant prostate cancer. It was not clear, however, whether the survival benefit was similar in all patients or whether some patients benefitted much more than others. The current study provides clear evidence that response to treatment with radium-223 varies markedly between patients. Whole-body DWI may therefore be useful in monitoring response during treatment with radium-223 and facilitate early stopping for lack of response. In addition, it will now be possible to test potential prognostic markers with respect to disease response on whole-body DWI. A small study suggested that the level of uptake on baseline fluoride-PET may predict radium-223 response (9). This finding is plausible because increased osteoblastic activity would be expected to increase uptake of both sodium 18F-fluoride and radium-223. This hypothesis can now be tested in men with metastatic castration-resistant prostate cancer using whole-body DWI to assess response.

Disease response to radium-223 varies markedly not just between patients but also between different metastases within the same patient. The reasons for this heterogeneity of response are unclear but will be important to understand to inform patient selection for treatment. It has been suggested in a mouse model of bone metastases that radium-223 is more effective against micrometastases and less effective against larger metastases (10). Whole-body DWI should enable us to address these issues for the first time in men with metastatic castration-resistant prostate cancer rather than in animal models. By contrast with the mouse model data, we observed the development of new bone metastases on DWI in 23 of 36 patients during treatment with radium-223, which suggests a lack of activity against micrometastases.

We observed an association between the administered activity of radium-223 and disease response on whole-body DWI. Higher administered activity was associated with a greater reduction in apparent diffusion coefficient in bone metastases present on baseline imaging. When using global median apparent diffusion coefficient, however, this association was no longer statistically significant. This apparent discrepancy may be explained, at least in part, by the appearance of new bone metastases during treatment. Thus, although there is a dose response for baseline lesions, patients are less likely to benefit from radium-223 dose escalation because of disease progression at other sites. This finding may explain the lack of benefit seen for radium dose escalation in a large, randomized phase 2 trial (11).

This study has some limitations. The sample size was chosen to analyze disease response on whole-body DWI, and the study was not powered to analyze overall survival in relation to radium-223 dose. New imaging modalities—notably, prostate-specific membrane antigen–PET—have become more widely used since the study was designed. The utility of prostate-specific membrane antigen–PET to assess response to radium-223 is not known. We acknowledge that whole-body DWI use is limited because of lack of MRI availability and radiologist expertise, but the increasing evidence for its multiple benefits, especially in patients with dominant bone disease, coupled with ongoing efforts toward technique standardization (both in acquisition and reporting) and software development for automatic reading are likely to accelerate its development.

In summary, whole-body DWI can be used to assess disease response in bone metastases. MRI response to treatment with radium-223 is heterogeneous, both between patients and between different metastases in the same patient. There is evidence for a dose-response relationship for radium-223. Disease progression can occur during treatment, with the development of new bone metastases. Therefore, an unmet need to identify pretreatment predictive markers of response to radium-223 remains.

## Supplementary Material

pkad077_Supplementary_Data

## Data Availability

The ICR-CTSU supports the wider dissemination of information from its research and increased cooperation between investigators. Trial data were collected, managed, stored, shared, and archived according to ICR-CTSU standard operating procedures to ensure the enduring quality, integrity, and utility of the data. Formal requests for data sharing are considered in line with ICR-CTSU procedures, with due regard given to funder and sponsor guidelines. Requests are through a standard proforma describing the nature of the proposed research and extent of data requirements. Data recipients are required to enter a formal data sharing agreement that describes the conditions for release and requirements for data transfer, storage, archiving, publication, and intellectual property. Requests are reviewed by the Trial Management Group in terms of scientific merit and ethical considerations, including patient consent. Data sharing is undertaken if proposed projects have a sound scientific or patient benefit rationale, as agreed to by the Trial Management Group and approved by the Independent Data Monitoring and Steering Committee, as required. Restrictions relating to patient confidentiality and consent will be limited by aggregating and anonymizing identifiable patient data. Additionally, all indirect identifiers that could lead to deductive disclosures will be removed in line with Cancer Research UK Data Sharing Guidelines.

## References

[1] Parker C , Nilsson S, Heinrich D, et al; ALSYMPCA Investigators. Alpha emitter radium-223 and survival in metastatic prostate cancer. N Engl J Med. 2013;369(3):213-223. 23863050 10.1056/NEJMoa1213755

[2] Scher HI , Halabi S, Tannock I, et al; Prostate Cancer Clinical Trials Working Group Design and end points of clinical trials for patients with progressive prostate cancer and castrate levels of testosterone: Recommendations of the Prostate Cancer Clinical Trials Working Group. J Clin Oncol. 2008;26(7):1148-1159. 18309951 10.1200/JCO.2007.12.4487 PMC4010133

[3] Padhani AR , Lecouvet FE, Tunariu N, et al METastasis reporting and data system for prostate cancer: practical guidelines for acquisition, interpretation, and reporting of whole-body magnetic resonance imaging-based evaluations of multiorgan involvement in advanced prostate cancer. Eur Urol. 2017;71(1):81-92. 27317091 10.1016/j.eururo.2016.05.033 PMC5176005

[4] Perez-Lopez R , Nava Rodrigues D, Figueiredo I, et al Multiparametric magnetic resonance imaging of prostate cancer bone disease: correlation with bone biopsy histological and molecular features. Invest Radiol. 2018;53(2):96-102. 28906339 10.1097/RLI.0000000000000415 PMC5768227

[5] Padhani AR , Liu G, Koh DM, et al Diffusion-weighted magnetic resonance imaging as a cancer biomarker: consensus and recommendations. Neoplasia. 2009;11(2):102-125. 19186405 10.1593/neo.81328 PMC2631136

[6] Hijab A , Curcean S, Tunariu N, et al Fracture risk in men with metastatic prostate cancer treated with radium-223. Clin Genitourin Cancer. 2021;19(5):e299-e305. 33958296 10.1016/j.clgc.2021.03.020 PMC8514085

[7] ISRCTN. *Radium-223: Evaluation of Activity and Surrogate Response 2015 [updated 07/02/2021]*. https://www.isrctn.com/ISRCTN17805587. Accessed September 25, 2023.

[8] Blackledge MD , Tunariu N, Orton MR, et al Inter- and intra-observer repeatability of quantitative Whole-Body, Diffusion-Weighted Imaging (WBDWI) in metastatic bone disease. PLoS One 2016;11(4):e0153840. 27123931 10.1371/journal.pone.0153840 PMC4849763

[9] Murray I , Chittenden SJ, Denis-Bacelar AM, et al The potential of (223)Ra and (18)F-fluoride imaging to predict bone lesion response to treatment with (223)Ra-dichloride in castration-resistant prostate cancer. Eur J Nucl Med Mol Imaging. 2017;44(11):1832-1844. 28612079 10.1007/s00259-017-3744-y PMC6175045

[10] Dondossola E , Casarin S, Paindelli C, et al Radium 223-mediated zonal cytotoxicity of prostate cancer in bone. J Natl Cancer Inst. 2019;111(10):1042-1050. 30657953 10.1093/jnci/djz007 PMC6792112

[11] Sternberg CN , Saad F, Graff JN, et al A randomised phase II trial of three dosing regimens of radium-223 in patients with bone metastatic castration-resistant prostate cancer. Ann Oncol. 2020;31(2):257-265. 31959342 10.1016/j.annonc.2019.10.025

