# Supplementary material for: Radium-223 in metastatic castration-resistant prostate cancer: whole-body diffusion-weighted magnetic resonance imaging scanning to assess response
Source: JNCI Cancer Spectr. 2023 Oct 3;7(6):pkad077. doi: 10.1093/jncics/pkad077 (PMC10640884; doi:10.1093/jncics/pkad077)
Supplement: pkad077_Supplementary_Data [file pkad077_supplementary_data.pdf]

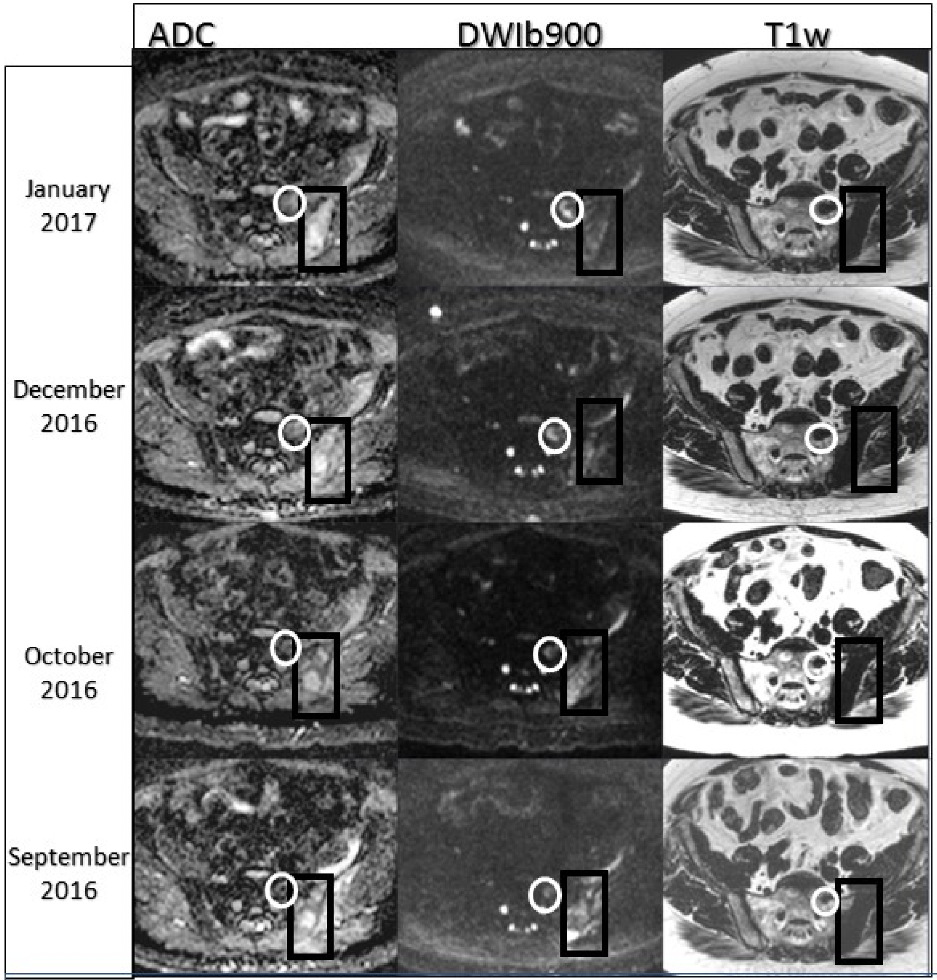

Supplementary Figure 1: Example of heterogeneous response to Radium 223:
Axial ADC , DWI b900 and T1W images though the pelvis demonstrating good response to Radium 223 in the left iliac bone metastasis (black square) as evidence by significant increase in ADC (50%). Note slow but unequivocal increase in size of the adjacent left S1 lesion (white circle) that shows heterogenous appearance on ADC with areas of low and high ADC suggestive of treated and active disease.
